# The iCre-DTA176 Mouse Exhibits Canonical Spontaneous Network Activity Associated With Retinal Degeneration

**DOI:** 10.1167/iovs.67.6.27

**Published:** 2026-06-15

**Authors:** Samuel William Fifield-Smith, Lay Khoon Too, Thomas Francis Cahir, Mohammad Hossein Khani, Matthew Patrick Simunovic, Dario Alejandro Protti

**Affiliations:** 1 Neuroscience Theme, School of Medical Sciences, The University of Sydney, Sydney, Australia; 2 Save Sight Institute, Faculty of Medicine and Health, The University of Sydney, Sydney, Australia; 3 Institute of Molecular and Clinical Ophthalmology Basel, Basel, Switzerland; 4 Department of Ophthalmology, University of Basel, Basel, Switzerland; 5 Sydney Eye Hospital, Sydney, Australia

**Keywords:** retinal degeneration, spontaneous retinal activity, network oscillations, gap junctions, optogenetic stimulation

## Abstract

**Purpose:**

Spontaneous rhythmic activity is a defining feature of degenerating retinas and poses a major barrier to effective vision restoration. In this study, we sought to determine the presence, underlying mechanisms and functional consequences of spontaneous network activity in the Rho-iCre-DTA176 mouse, a novel model of retinal degeneration.

**Methods:**

Extracellular recordings were obtained from isolated retinas using multielectrode arrays to characterize spontaneous and optogenetically evoked retinal ganglion cell (RGC) activity. Network mechanisms were probed pharmacologically by disrupting electrical coupling using the gap-junction blocker meclofenamic acid (MFA).

**Results:**

Retinal ganglion cells in Rho-iCre-DTA176 retinas exhibited pronounced oscillatory burst firing characterized by short interspike intervals, high burst occupancy and narrowband spectral structure. MFA selectively reduced short interspike intervals and abolished rhythmic bursting activity while sparing residual spontaneous spiking, thus supporting a network-driven origin of the aberrant activity. At the functional level, suppressing spontaneous oscillations significantly improved the signal-to-noise ratio of optogenetically evoked responses.

**Conclusions:**

These results demonstrate that pathological retinal oscillations in the Rho-iCre-DTA176 mouse are driven by gap-junction–dependent network mechanisms and closely resemble those observed in established retinal degeneration models. Together, the results validate the Rho-iCre-DTA176 mouse as a valuable retina degeneration model for evaluating strategies aimed at restoring visual function.

Inherited retinal degeneration (IRD)—a diverse group of retinal diseases that affect millions of individuals worldwide—are characterized by a progressive loss of photoreceptor function, leading to vision impairment and ultimately blindness.^1^^–^^3^ Understanding the genetic basis and pathophysiological mechanisms of these diseases is critical for developing effective causative gene-specific and gene-agnostic treatments aimed at restoring vision.^3^^–^^5^ In this context, rodent models of retinal degeneration, have been pivotal in advancing our current understanding of retinal degeneration because of their ease of genetic manipulability, relatively low maintenance cost and rapid disease progression.^6^^–^^8^

The rd_1_ and rd_10_ mouse models have been extensively used to study rod-specific degenerations and test therapeutic interventions for retinal diseases. Studies on the rd_1_ mouse model revealed that a mutation in the phosphodiesterase 6B gene leads to accumulation of cyclic GMP in rod photoreceptors, causing their death and thinning of the outer nuclear layer.^9^^,^^10^ Comparative analyses of rd_1_ and rd_10_ mice demonstrated the progression and timing of photoreceptor loss, identifying key periods for intervention, thereby informing the development of potential treatment strategies.^6^ Recent developments in retinal therapeutic interventions using rd_1_ and rd_10_ mouse models include the use of iPSC-derived retina cellular transplants, which have shown promise in restoring visual function by developing mature outer nuclear layers and restoring light responses.^11^^,^^12^ Further studies using rd_1_ mouse models have also shed light into the neuroprotective properties of various pharmacological interventions. For example, the use of cGMP analogues encapsulated within nanosized liposomal delivery systems has shown promising results in preserving retinal function and protecting against photoreceptor loss.^13^ Additionally, repurposing of the biguanide, metformin, which is primarily used in Type II diabetes, reduces apoptosis in photoreceptors and suppresses microglial activation in the outer nuclear layer.^14^ Other rodent models, including the Tubby (rd_5_) mouse,^15^ RBF/DnJ rd_3_ rat,^16^ Fischer 344 rat,^17^ and Royal College of Surgeons rat^18^ provide valuable tools for investigating the genetic and physiological aspects of retinal degenerations.^6^^,^^19^^,^^20^ Together, these studies highlight the invaluable role of animal models in advancing our understanding and the development of novel treatments for IRD.

Photoreceptor death not only compromises visual perception by reducing “signal” but also increases “noise” via significant changes within the retinal circuitry, leading to the emergence of spontaneous rhythmic activity in RGCs.^21^^–^^23^ This phenomenon has been consistently documented across numerous rodent models of retinal degeneration, suggesting a unified pathological mechanism.^23^^,^^24^ This activity has been hypothesized to reflect the maladaptive plasticity of the retinal network in response to sensory deafferentation, yet its precise mechanisms remain unknown.^25^^,^^26^ The use of rd_1_ and rd_10_ mice has been instrumental in studying the origin of spontaneous rhythmic activity in the degenerate retina. Pharmacological interventions have shed light on the biophysical basis of the oscillations in the degenerate retina, emphasizing the role of gap junction-mediated network interactions in sustaining so-called “pacemaker activity” within the AII amacrine–ON cone bipolar cell network.^27^^,^^28^ AII amacrine cells are electrically coupled amongst themselves and to ON cone bipolar cells via gap junctions^29^ and were shown to facilitate synchronized activity across the retina.^28^^,^^30^ Blockade of gap junctions with meclofenamic acid (MFA) disrupts oscillatory rhythmic activity.^26^^,^^28^^,^^31^ In addition, voltage-gated Na^+^ channels were shown to also drive and synchronize intrinsic oscillations.^28^ Because disease-induced oscillations are predicted to degrade retinal signal processing, they may similarly limit the effectiveness of vision restoration therapies. Thus understanding the nature and origin of oscillatory activity in retinal degeneration is critical for developing targeted treatments, optimizing vision restoration and improving patient outcomes.

The Rho-iCre-DTA176 mouse, a novel model of retinal degeneration, designed to replicate some of the pathophysiological characteristics of retinal degenerative diseases via the targeted ablation of rod photoreceptors. In this model, activation of the diphtheria toxin fragment A (DTA176) gene expression via the rhodopsin (rho) promoter leads to early onset, severe retinal degeneration characterized by the loss of the outer nuclear layer by weaning age.^32^ Moreover, the Rho-iCre-DTA176 model shows a complete absence of electroretinographic responses, thus validating its use in modeling the functional deficits observed in retinal degeneration.^32^ Consequently, this model offers a valuable tool for exploring therapeutic strategies aimed at vision restoration in degenerative retinal conditions.

In this study, we aimed to firstly determine whether or not the iCre-DTA176 model displays degeneration-mediated spontaneous rhythmic activity similar to that observed in established retinal degeneration models, and secondly assess its validity for studying retinal degeneration mechanisms and associated recovery of typical photoreceptors-driven retinal electrical activity using optogenetic treatment. We characterized the cellular dynamics that govern spontaneous rhythmic activity and its pharmacological sensitivity and found that Rho-iCre-DTA176 retinas exhibit robust, oscillatory burst spiking driven by gap junction-coupled networks, and that pharmacological suppression of this hyperactivity significantly enhances the signal-to-noise ratio of optogenetically evoked responses. Our findings validate the relevance of the Rho-iCre-DTA176 animal model for retinal degeneration research and the development of novel therapeutic interventions.

## Methods

### Animals and Generation of the Dystrophic Mouse Model

The Rho-iCre-DTA176 mouse model of retinal degeneration was generated as previously described^32^ by crossing rhodopsin-Cre mice^33^ with Rosa-DTA176 mice,^34^ resulting in selective expression of diphtheria toxin fragment A (DTA176) in rod photoreceptors. This genetic strategy produced targeted ablation of photoreceptors in Cre-positive offspring. By the time of weaning (postnatal day 21–28), retinal dystrophic mice exhibited complete loss of the outer nuclear layer, accompanied by the absence of detectable electroretinographic responses, consistent with advanced retinal degeneration. Age- and sex-matched wild-type (WT) littermates were used as controls. WT and dystrophic mice of both sexes, aged six to 18 months, were group-housed together (two to six animals per cage) under temperature-controlled conditions and a 12-hour light/12-hour dark cycle, with *ad libitum* access to standard food and water. Bilateral intravitreal injections of adeno-associated viral 2, 7m8 variant (AAV2.7m8) vectors were performed in dystrophic mice at two months of age following the procedures described by Too et al.^32^ Dystrophic mice were randomly assigned to experimental groups and received bilateral intravitreal injections of AAV2.7m8 carrying either the treatment construct *CamkIIα* -bReaChES-TS-eYFP) or control construct that lacked the sequence for bReaChES (*CamkIIα*-eYFP). All experimental procedures involving animals were approved by The University of Sydney Animal Ethics Committee and adhered to the guidelines for animal experiments issued the NSW Animal Research Act (1985—Animal Research Regulation 2010) and the 2013 NHMRC “Australian code for the care and use of animals for scientific purposes” and the ARVO Statement for the Use of Animals in Ophthalmic and Vision Research.

### Intraocular Injections

Bilateral intravitreal injections of AAV vectors were performed in two-month-old mice. Animals were anesthetized by intraperitoneal injection of ketamine (38.4 mg/kg) and medetomidine (0.48 mg/kg), and pupils were dilated with topical 1% tropicamide and 2.5% phenylephrine. Under a dissecting microscope (Leica M60; Leica Microsystems, Wetzlar, Germany), 2 µL of viral suspension was injected into each eye using a 32-gauge type 4 needle (Hamilton Company, Reno, NV, USA) attached to a 5-µL Hamilton syringe (model 65). The optogenetic vector rAAV2/*CamkIIα*-bReachES-TS-eYFP or the rAAV2/*CamkIIα*-ChRmine-eYFP was delivered at a titer of 2 × 10¹²-1 × 10¹³ viral genomes/mL, as described previously.^35^ Anesthesia was reversed with intraperitoneal atipamezole (0.96 mg/kg).

Multielectrode array (MEA) recordings were obtained from isolated retinas of six retinas from five wild-type mice, five retinas from five Rho-iCreDTA176 mice that did not receive viral injections, and nine retinas from nine Rho-iCreDTA176 mice injected with an opsin-expressing virus, as previously described. Wild-type retinas were used as baseline controls for retinal network activity, whilst untreated age- and sex-matched Rho-iCreDTA176 retinas served as a control for effects of genetic background and transgene expression independent of viral manipulation. Comparisons between untreated and treated (control and treatment vectors) retinas allowed separation of baseline spontaneous activity from virus- and opsin-mediated effects. Inclusion of both transgenic groups also controlled for nonspecific effects of the injection procedure.

### Fundus Imaging

Four to six weeks after intravitreal injection, in vivo fundus imaging was performed to assess retinal integrity and transgene expression as described in Too et al.^32^ Briefly, mice were anesthetized by intraperitoneal administration of ketamine (38.4 mg/kg) and medetomidine (0.48 mg/kg), and pupils were dilated with topical 1% tropicamide and 2.5% phenylephrine. Fundus images were acquired using a MICRON IV retinal imaging system (Phoenix Technology Group, Lakewood, CO, USA) in brightfield and fluorescence modes to confirm transgene expression. Anesthesia was reversed by intraperitoneal injection of atipamezole (0.96 mg/kg).

### Genotyping

Transgenic animals were genotyped by PCR using a three-primer strategy. Primer WS268 (forward; GTT ATC AGT AAG GGA GCT GCA GTG G) together with WS270 (wild-type reverse; AAG ACC GCG AAG AGT TTG TCCTC) amplified a 415-bp fragment corresponding to the wild-type ROSA26 allele, whereas WS268 together with WS271 (DTA176-specific reverse; GGC GGA TCA CAA GCA ATA ATA ACC) amplified a 302-bp fragment corresponding to the ROSA-DTA176 allele. PCR conditions were: 94°C, 90 seconds, 36 cycles (94°C 30 seconds; 59°C, 45 seconds; 72°C, 60 seconds), 72°C, 10 minutes.

### Tissue Preparation and Extracellular Recordings

Animals were euthanized by cervical dislocation and both eyes were rapidly enucleated and transferred to carboxygenated Ames medium (95% O₂/5% CO₂; Sigma-Aldrich Corp., St. Louis, MO, USA). The cornea, lens and vitreous humor were carefully removed. The eyecup was bisected, a hemiretina was gently separated from the retinal pigment epithelium, mounted ganglion cell-side down onto a multielectrode array (MultiChannel Systems, Reutlingen, Germany; 60-electrode perforated arrays; 30 µm electrode diameter; 200 µm interelectrode spacing) transferred to an upright microscope (BX50 WI; Olympus Corp., Tokyo, Japan). The remaining retinal tissue was stored in carboxygenated Ames medium for subsequent recordings.

During recordings, the retina was continuously perfused with carboxygenated Ames medium at 4–5 mL/min, and the temperature of the recording chamber was maintained at ∼35°C using an inline heater (TC-344B; Warner Instruments, Holliston, MA). Signals from the RGCs were amplified, band-pass filtered (300 Hz to 5 kHz) and stored digitally using an Intan 512 Ch RHD recording system (Intan Technologies; Los Angeles, CA, USA) at a sampling rate of 20 kHz. Recordings were continuous for each experimental condition to preserve long-range temporal structure in spontaneous and evoked activity.

Light stimulation was delivered as full-field illumination through the microscope optics using a 4× objective and a 565-nm LED (bandwidth Δλ = 104 nm; Thorlabs, Newton, NJ, USA). Stimuli were presented at three different durations with an irradiance of 15.5 log photons · cm⁻² · s⁻¹. Light pulses were controlled and synchronized by an LIH 8+8 Data Acquisition Interface operated via PatchMaster (HEKA Elektronik, Lambrecht, Germany).

### Spike Detection and Sorting

Spike detection and sorting were performed using a modified version of Kilosort.^36^^,^^37^ The original version is available at the MouseLand repository (https://github.com/MouseLand/Kilosort), and the MEA-adapted version developed by Karamanlis and Khani is available from the KiloSortMEA repository (https://github.com/dimokaramanlis/KiloSortMEA). Kilosort outputs were visually inspected and manually curated using Phy2 (https://github.com/cortex-lab/phy) to refine unit classification and exclude artifacts. Units were classified as single units based on waveform consistency, refractory period violations, and other metrics. Only well-isolated units were included in all subsequent analyses. Spike times were exported as discrete time stamps for each unit.

### Inter-Spike Interval Analysis and Burst Definition

Spike trains were analyzed to quantify bursting structure and temporal dynamics. For each unit, inter-spike intervals (ISIs) were computed as:

$$ISI_{i}=t_{i+1}-\;t_{i}$$

where *t_i_* denotes the time of the i^th^ spike.

Bursts were defined using a threshold-based ISI criterion. A burst was identified as a sequence of at least four consecutive spikes with all inter-spike intervals shorter than 40 ms and a total burst duration of at least 60 ms. Burst onset was defined as the time of the first spike in the sequence and burst offset as the last spike satisfying the criteria. For each unit, the following burst metrics were quantified:

Burst duration:

$$D_{burst}=t_{last}-\;t_{first},$$

where *t_first_* and *t_last_* are the times of the first and last spikes in a burst.

Burst frequency:

$$f_{burst\;}=\frac{N_{bursts}}{T},$$

where *N_bursts_* is the total number of detected bursts and *T* is the recording duration.

Intra-burst firing frequency:

$$f_{intra\;}=\frac{N_{spikes,burst}-1}{D_{burst}},$$

computed for each burst and averaged per unit.

Total firing rate:

$$f_{total}\;=\frac{N_{spikes}}{T},$$

Burst spike percentage:

$$P_{burst}=\frac{N_{spikes,burst}}{N_{spikes,total}}\;\times \;100,$$

representing the fraction of all spikes occurring within bursts.

### Temporal Heatmaps and Occupancy

To visualize population-level temporal structure, burst activity was summarized in time-binned heatmaps. Time was discretized into bins of fixed width, and for each bin the number of bursts per unit was counted. Heatmaps were constructed with units along one axis and time along the other to reveal temporal clustering and rhythmic structure.

Population occupancy was computed as a measure of synchrony across units. For each time bin, occupancy was defined as:

$$Occupancy_{\left(t\right)}=\frac{N_{active\;units\;}\left(t\right)}{N_{total\;units}},$$

where an active unit was defined as one exhibiting at least one burst within the bin.

### Spectral Analysis of Burst Dynamics

Spectral analyses were performed on the temporal sequence of burst onsets. Burst onset times were converted into a discrete-time signal by binning burst counts per time bin, yielding a burst-onset sequence.

Time-frequency structure was analyzed using the short-time Fourier transform. The burst-onset sequence was segmented into overlapping windows of two seconds’ duration, shifted in steps of 0.25 s. For each window, a Fourier transform was computed to estimate the power spectral density. Spectral estimates from successive windows were concatenated to generate a time-frequency spectrogram.

### Spectral Flatness

Spectral flatness, a metric used in audio engineering and auditory neuroscience,^38^^,^^39^ was used to quantify the degree to which power spectra were dominated by narrowband oscillations versus broadband activity. For each spectral window, flatness was calculated as:

$$Spectral\;Flatness=\frac{Geometric\;mean\;\left(P_{x}\right)}{Arithmetic\;mean\;\left(P_{x}\right)},$$

where *P_x_* are the power spectral values. Values near zero indicate peaky, oscillatory spectra, whereas values near one indicate spectrally flat activity.

### Signal-to-Noise Ratio (SNR)

Signal-to-noise ratio was used to quantify the separability of stimulus-evoked responses from spontaneous activity. For each unit, evoked spike counts were measured across 100 trials per stimulus duration within a 10 ms post-stimulus window for 1 and 3 ms stimuli, and within a 16 ms window for 10 ms stimuli. Spontaneous activity was estimated by randomly sampling baseline windows of identical duration from stimulus-free periods using a bootstrapping procedure (100,000 resampling iterations per unit and condition). SNR was computed as:

$$SNR=\frac{\mu_{s}-\mu_{n}}{\sigma_{n}},$$

where µ_*s*_ denotes the mean evoked spike count (signal + noise), µ_*n*_ the mean spontaneous spike count, and σ_*n*_ the standard deviation of spontaneous spike counts.

### Statistical Analysis

Data analysis was performed using custom-written routines in IGOR (Wavemetrics, Lake Oswego, OR, USA) and Python (v3.9). Statistical analyses were performed using Python and GraphPad Prism (Boston, MA, USA). Group comparisons of burst metrics, occupancy and spectral measures were performed using non-parametric statistical tests. Differences across multiple groups were assessed using Kruskal–Wallis tests, followed by pairwise Mann–Whitney U tests. Bonferroni correction was applied to control for multiple comparisons. Statistical significance was defined as (*P* < 0.05).

## Results

### Rho-iCre-DTA176 Retinas Exhibit Abnormal Spontaneous Firing Patterns

**Figure 1.**
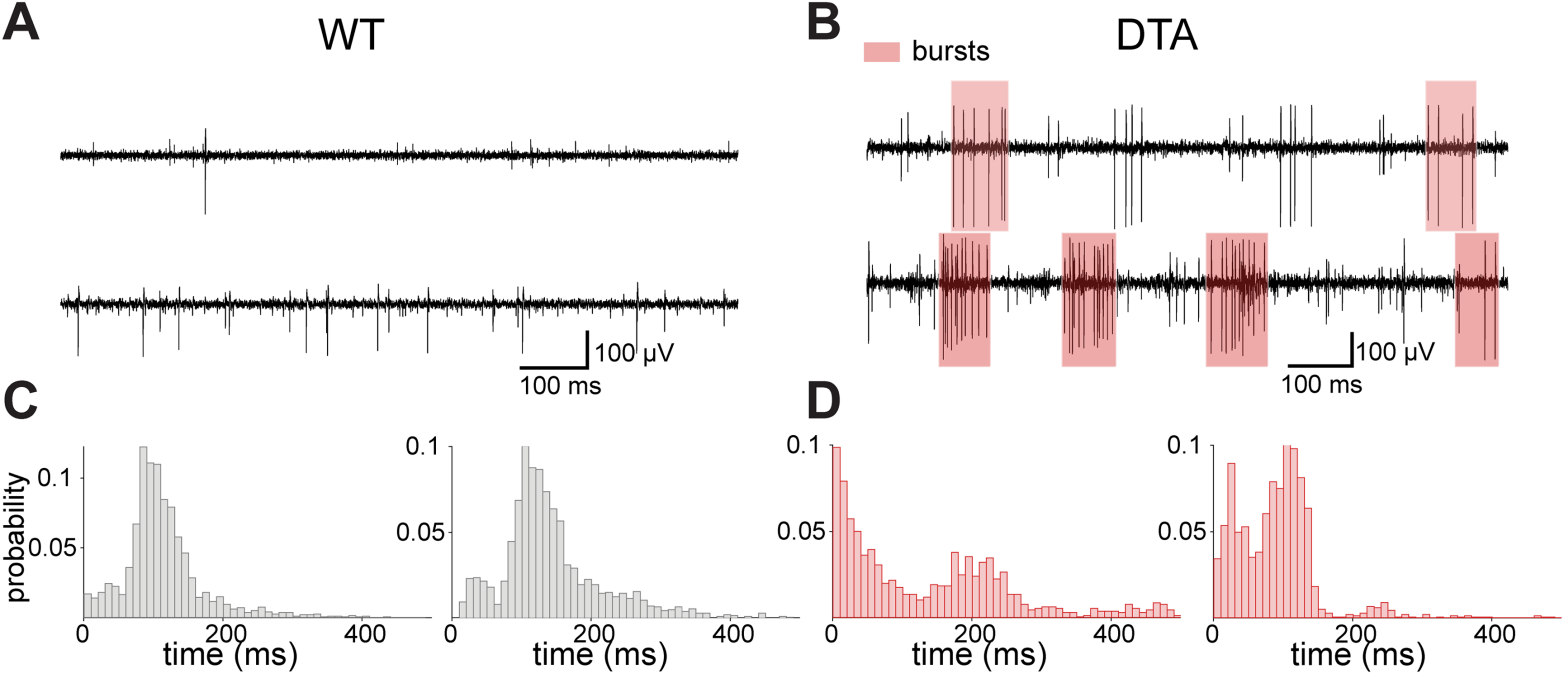
Spontaneous activity and interspike intervals in wild-type and Rho-iCre-DTA176 mice. **(A, B)** Representative extracellular traces from wild-type **(A)** and Rho-iCre-DTA176 **(B)** retinas. Rho-iCre-DTA176 recordings display frequent bursts (highlighted in *red*), absent in wild-type. **(C, D)** Interspike interval distributions. WT shows longer intervals with a unimodal distribution, whereas Rho-iCre-DTA176 displays shortened ISIs and burst-associated peaks.

To examine whether or not photoreceptor loss in the DTA mouse model modifies spontaneous activity in RGCs, we first compared baseline spontaneous spike trains in WT and DTA176 retinas. Our initial goal was to determine whether the DTA176 mutation alters RGC spiking, as many IRD models show a transition from sparse firing to clustered hyperactivity. Figures 1A and 1B show representative extracellular traces that illustrate this shift in the basic temporal organization of RGC spontaneous activity, with units from WT retinas discharging irregularly with long intervals between spikes, whereas DTA176 units displayed prominent, recurrent bursts highlighted in red.

To evaluate these differences, we next examined ISI distributions. Figure 1C shows that, for the same unit as in Figure 1A, WT ISIs were unimodally distributed and dominated by long intervals, whereas ISIs from DTA176 (same unit as in Fig. 1B) exhibited multiple short-interval peaks consistent with burst firing (Fig. 1D). This substantial compression of ISIs indicates that Rho-iCre-DTA176 retinas display a distinct firing regime in which spikes cluster into high-frequency episodes rarely seen in wild-type retinas. Taken together, these data suggest that retinal degeneration secondary to DTA-associated photoreceptor expression induces a transition from sparsely distributed spikes to pronounced bursting, providing evidence that spontaneous network activity is fundamentally altered in this pre-clinical IRD model.

### Rho-iCre-DTA176 Retinas Display Increases In Frequency, Duration, and Spike Count of Bursts Compared to WT Animals

**Figure 2.**
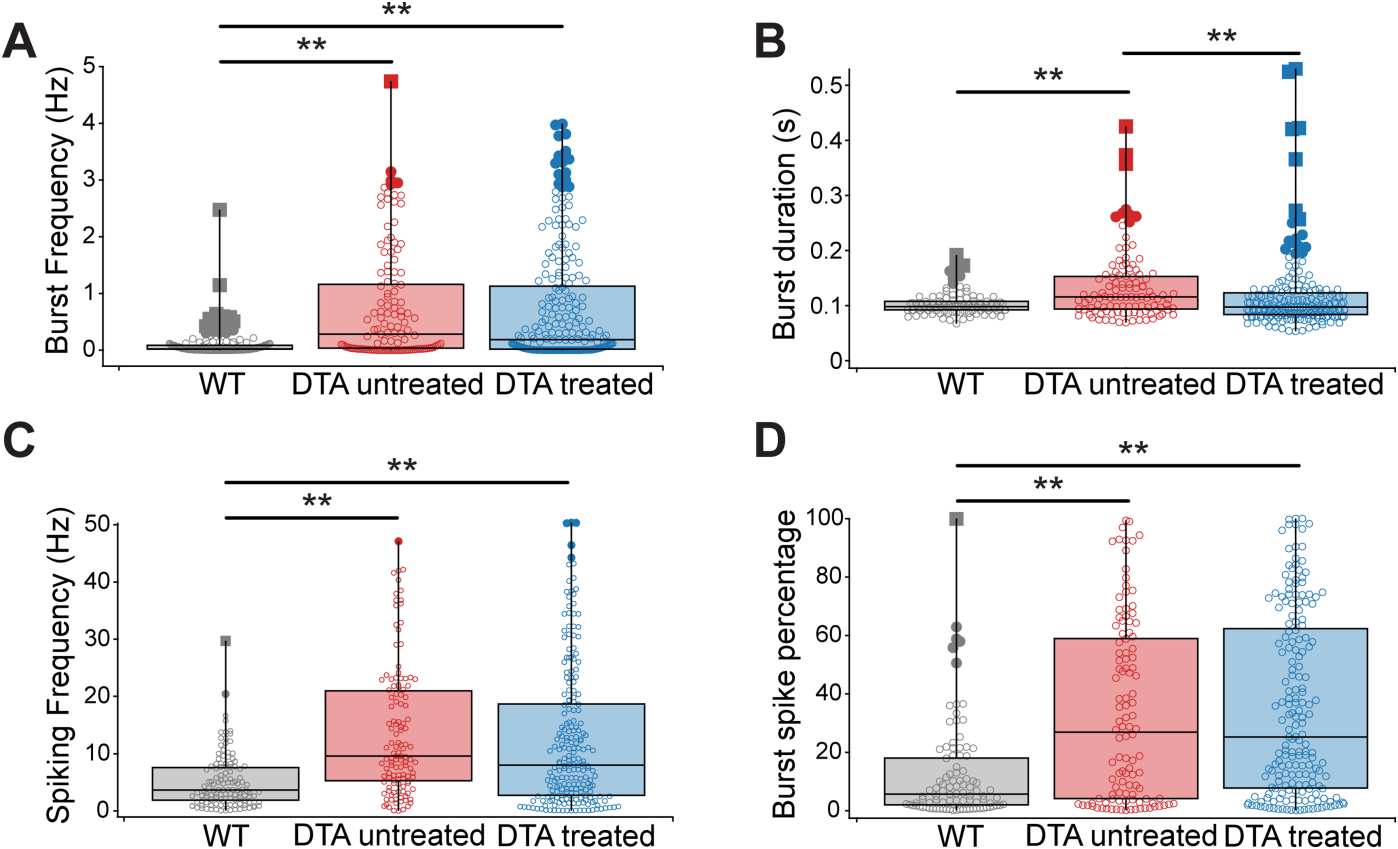
Rho-iCre-DTA176 mouse retinas display increased bursting and spiking activity. **(A)** Boxplots of burst frequency show significantly higher values in Rho-iCre-DTA176 compared to WT. **(B)** Burst duration is longer in Rho-iCre-DTA176 untreated compared to WT and Rho-iCre-DTA176 treated retina. **(C)** Spiking frequency is markedly increased in Rho-iCre-DTA176 retinas. **(D)** Burst spike percentage is elevated in DTA176 compared to WT. WT: 122 units, seven retinas, Rho-iCre-DTA176 untreated: 125 units, five retinas, Rho-iCre-DTA176 treated: 224 units, nine retinas. (*P* < 0.01, Mann-Whitney U test).

To quantify the degree to which bursting differed across genotypes and treatments, we measured burst frequency, duration, spiking rate, and burst spike percentage in WT, untreated Rho-iCre- and treated Rho-iCre-DTA176 treated retinas. We first determined whether DTA176 retinas simply exhibit more bursts. As shown in Figure 2A, burst frequency was significantly increased in both Rho-iCre-DTA176 retinas relative to WT (Mann-Whitney U, *P* < 0.01), with untreated mutants showing the strongest effect.

We then conducted a systematic analysis of burst duration. Figure 2B shows that bursts in untreated Rho-iCre-DTA176 retinas lasted significantly longer than in both WT and treated Rho-iCre-DTA176 retinas (*P* < 0.01), indicating that IRD in the Rho-iCre-DTA176 model leads to prolonged bursts of high-frequency firing. Next, we assessed overall spiking frequency (Fig. 2C), finding significantly increased rates in both treated and untreated Rho-iCre-DTA176 retinas (*P* < 0.01), consistent with the emergence of sustained hyperactivity. Finally, we quantified the proportion of spikes occurring within bursts. Figure 2D indicates that DTA176 retinas allocate a substantially greater fraction of their total spiking to burst epochs. These findings show that the Rho-iCre-DTA176 retinas phenotype reflects not only an increase in burst count but an increase in all burst-associated metrics resulting in a dominant bursting mode that shapes most of the spontaneous output.

### Rho-iCre-DTA176 Retinas Show Elevated Burst Occupancy and Altered Temporal Organization

**Figure 3.**
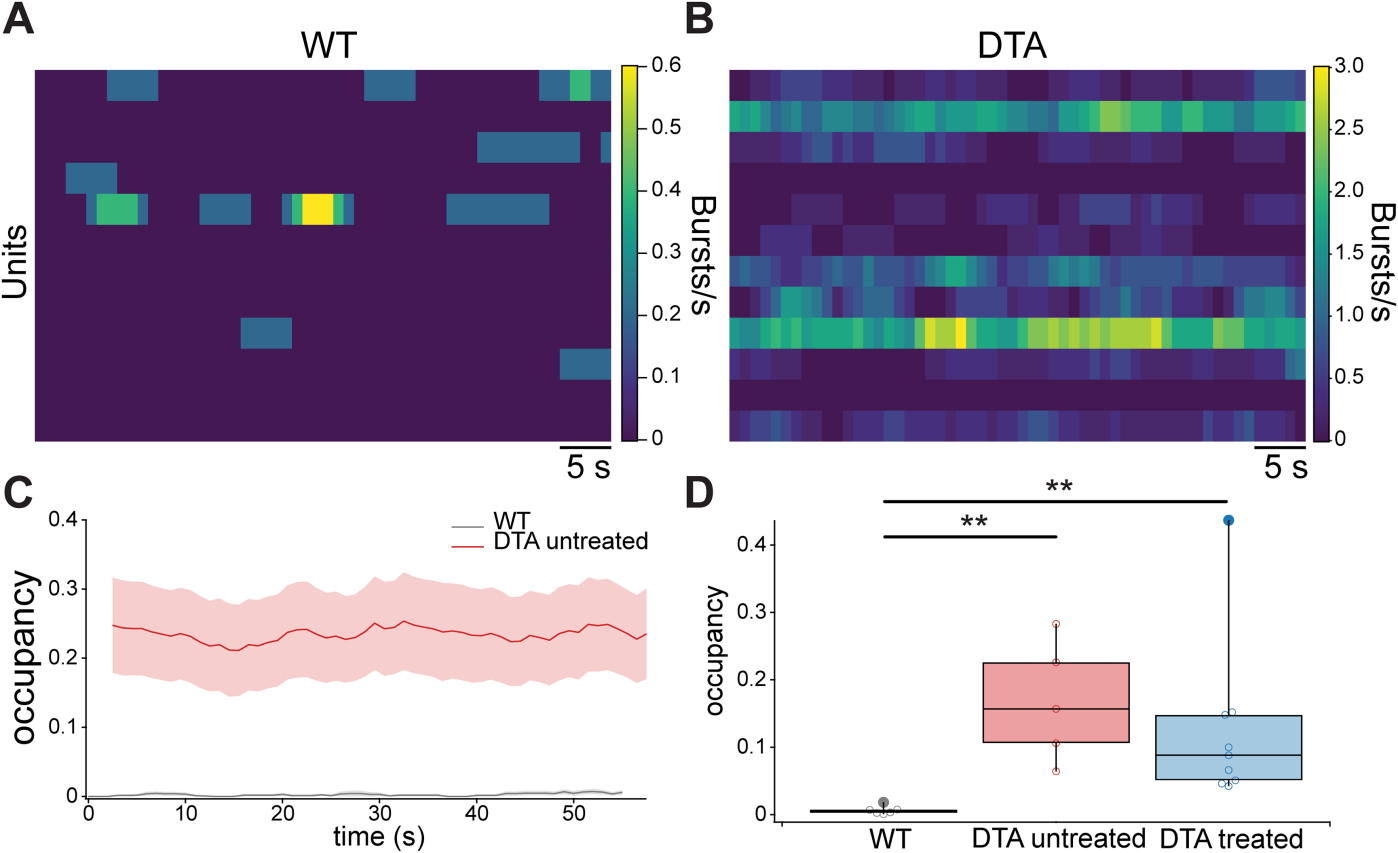
Increased burst activity and occupancy in Rho-iCre-DTA176 mice. Heatmaps of burst activity over time for individual units in wild-type **(A)** and Rho-iCre-DTA176 **(B)** retinas. Each row represents an individual RGC (units). **(C)** Mean occupancy across time shows consistently elevated activity in Rho-iCre-DTA176 retina compared to WT. *Shaded area*: SEM. **(D)** Boxplots of mean occupancy per group. Rho-iCre-DTA176 (both treated and untreated) mice show significantly higher occupancy than WT (*P* < 0.01, Mann-Whitney U test).

To assess how these changes scale to the level of the RGC population, we examined burst occupancy across simultaneously recorded units. We first evaluated how individual units contribute to population-wide dynamics. Figures 3A and 3B show heatmaps of individual experiments in which WT retinas exhibit sparse burst activity distributed over time, whereas Rho-iCre-DTA176 retinas display high frequency, recurring windows of synchronous bursting in a much larger proportion of units than WT retina. To systematically investigate the effect of photoreceptor loss on ensemble behavior, we extracted the mean occupancy across time. Figure 3C shows that Rho-iCre-DTA176 retinas maintained consistently higher occupancy than WT, with large oscillatory fluctuations characteristic of network-level synchrony. Quantification across all units in Figure 3D confirmed significantly elevated mean occupancy in both untreated and treated Rho-iCre-DTA176 retinas compared to WT retinas (Mann-Whitney U; *P* < 0.01). These results indicate that hyperactivity in Rho-iCre-DTA176 retinas is not limited to single-unit firing abnormalities but reflects increased activity spanning broad neuronal ensembles.

### Spectral Analyses Reveal Structured Oscillatory Activity in Rho-iCre-DTA176 Retinas

**Figure 4.**
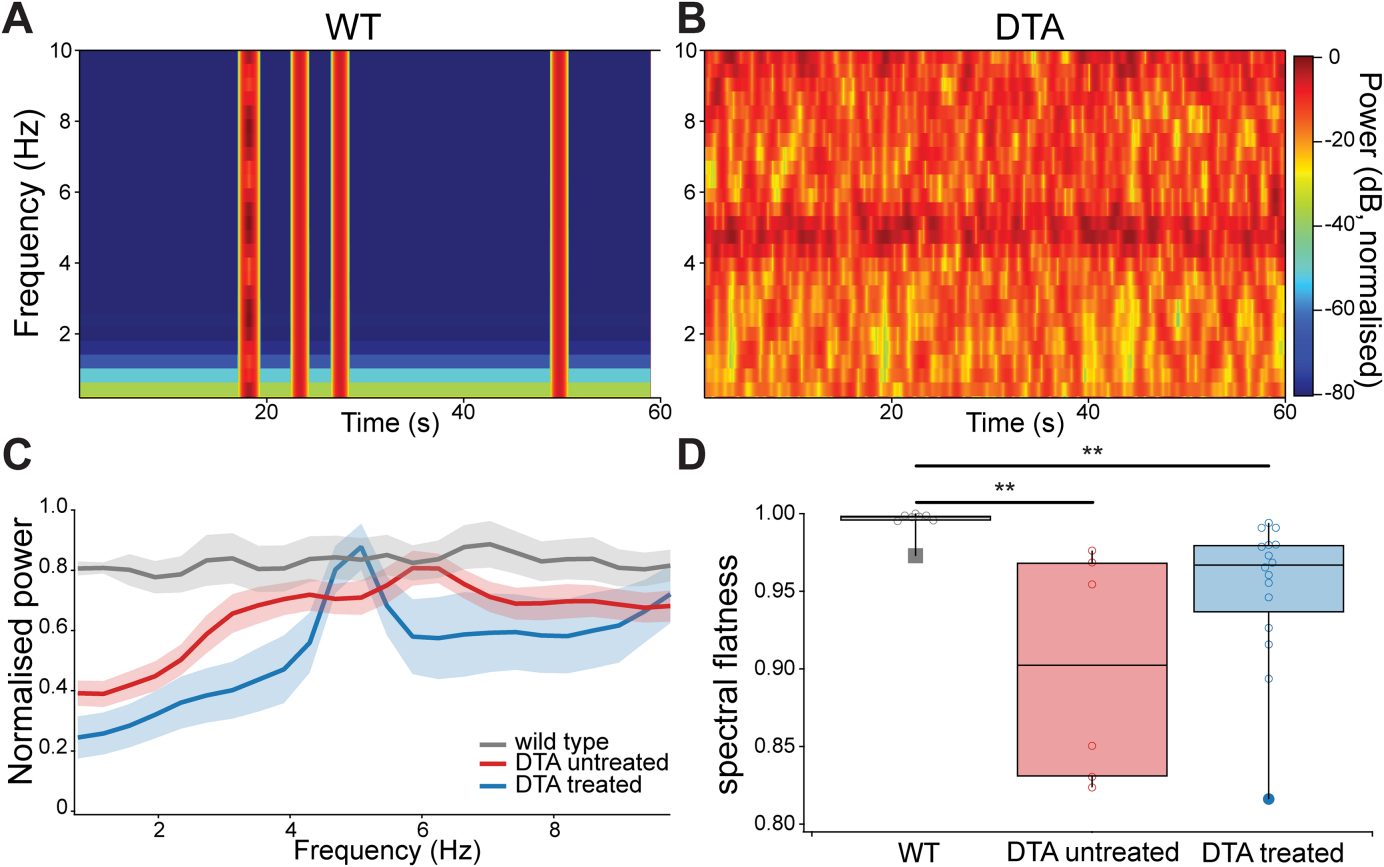
Spectral properties of burst activity in wild-type and Rho-iCre-DTA176 retinas. **(A, B)** Spectrograms showing time-varying frequency content in representative RGCs. **(C)** Normalized power spectra reveal distinct frequency peaks in Rho-iCre-DTA176 mice compared to flattened spectra in WT. *Shaded areas*: SEM. **(D)** Boxplots of spectral flatness demonstrate significantly higher flatness in WT compared to Rho-iCre-DTA176 (*P* < 0.01, Mann- Whitney U test).

The emergence of pathological, rhythmic hyperactivity is widely attributed to the unmasking of intrinsic oscillators within electrically coupled interneuron networks, which drive synchronous synaptic input to RGCs in the 5–10 Hz frequency band. To evaluate the presence and spectral characteristics of such oscillatory drive within the Rho-iCre-DTA176 retinas, we performed a time-frequency analysis of burst-onset sequences. Figures 4A and 4B show spectrograms from representative cells where it can be observed that WT RGCs exhibited largely flat, low-power spectra interrupted by irregular transients, whereas Rho-iCre-DTA176 RGCs showed sustained rhythmic activity with clear harmonic structure, in this case in the ∼5 Hz range.

We next examined normalized power spectra across groups. As shown in Figure 4C, WT spectra remained shallow across 2–10 Hz, while DTA176 spectra were dominated by pronounced 2–6 Hz peaks. To assess whether burst power was distributed uniformly across spectral bands or concentrated within a narrower subset, we quantified the spectral shape using spectral flatness. We found significantly higher spectral flatness values in WT than in DTA176 (Fig. 4D; *P* < 0.01), consistent with a change from broadband, noise-like activity in the WT retina to more organized and rhythmic spectral structure in the Rho-iCre-DTA176 retina. These findings suggest that Rho-iCre-DTA176 retinas display a robust oscillatory generator consistent with gap-junction-driven pacemaking, establishing a mechanistic parallel with classical IRD models.

### Gap Junction Blockade With MFA Suppresses Bursting Activity

**Figure 5.**
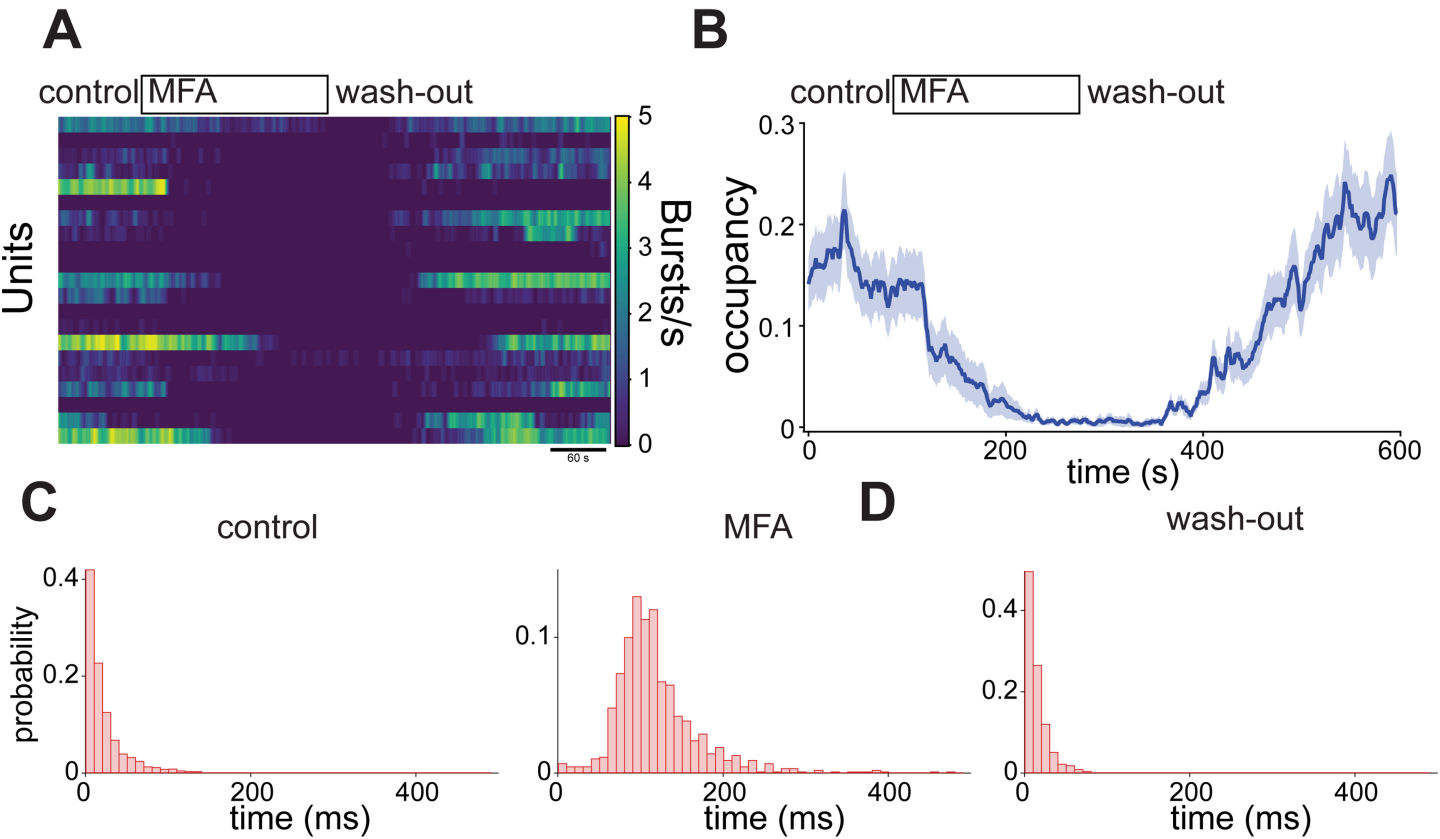
Effects of MFA on burst dynamics and inter-spike intervals in Rho-iCre-DTA176 retinas. **(A)** Heatmap of burst temporal dynamics across units during control, 50 µM MFA application, and washout. **(B)** Population occupancy traces over time, showing reduced occupancy during MFA that recovers after washout. *Shaded area*: SEM. **(C, D)** ISI distributions under control, MFA, and washout conditions. MFA reduces the probability of short ISIs, consistent with reduced bursting, which returns toward baseline after washout.

To evaluate whether bursting in the Rho-iCre-DTA176 model arises from gap junction-coupled networks, particularly those involving AII amacrine cells, like in other pre-clinical IRD models, we tested whether the gap junction blocker MFA could attenuate burst dynamics. First, we established baseline conditions by recording spontaneous burst activity and then we applied 50 µM MFA to block electrical coupling. Heatmaps in Figure 5A show that MFA strongly reduced burst occurrence across all units and this suppression was reversed during after washout. Population occupancy traces (Fig. 5B) dropped to almost zero during MFA application and rebounded after washout.

MFA abolished burst occurrence and strongly reduced, but did not completely eliminate, spontaneous spiking activity. To determine how these changes were expressed at the level of basic firing structure, we compared ISI distributions across control, MFA and washout conditions. Several different outcomes were possible, each reflecting a distinct underlying mechanism. A shift toward longer ISIs would suggest that gap-junction blockade disrupts the oscillatory network responsible for generating closely clustered spikes without fully suppressing intrinsic spiking activity, whereas little or no change in ISIs distribution would imply a general reduction in overall firing without altering their temporal pattern. Conversely, an increase in short intervals would suggest that gap junctions normally exert an inhibitory influence on spiking activity. Figures 5C and 5D show that MFA greatly reduced the probability of short ISIs and shifted the distribution toward longer intervals, a pattern consistent with substantially weakened burst expression, and this effect was reversed by washout. These findings suggest that gap-junction coupling is essential for sustaining Rho-iCre-DTA176 burst dynamics, and pharmacological block disrupts both the frequency and synchrony of pathological activity.

### MFA Enhances the Signal-to-Noise Ratio of Optogenetically Evoked Responses

**Figure 6.**
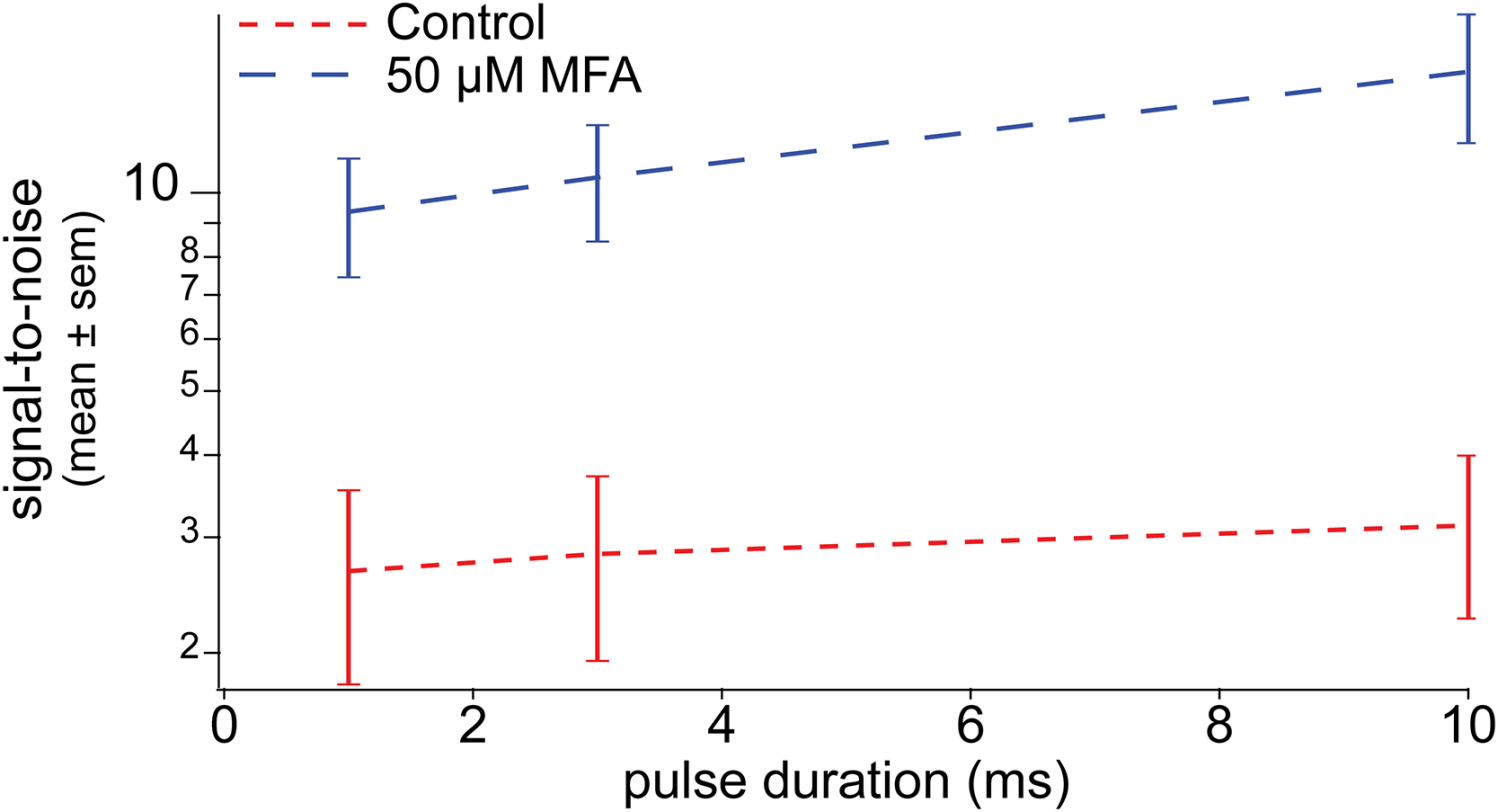
MFA enhances signal-to-noise ratio of optogenetically evoked responses. Signal-to-noise ratio (mean ± SEM) in Rho-iCre-DTA176 retinas as a function of pulse duration under control conditions (*red*) and in the presence of 50 µM MFA (*blue*). MFA significantly increased SNR across all pulse durations compared to control. 20 units, two retinas.

This hyperactivity severely compromises visual signaling fidelity and poses a critical challenge for vision restoration strategies, as elevated background noise can obscure the precise temporal features of optogenetically driven responses. Previous work indicates that pharmacological blockade with MFA helps recover signal fidelity. Building on these findings, we sought to determine whether pharmacologically silencing aberrant spontaneous noise in the Rho-iCre-DTA176 model similarly enhances the detectability of optogenetically evoked signals. To assess the impact of spontaneous bursting on optogenetically driven responses, we quantified the SNR under control and MFA conditions in Rho-iCre-DTA176 retinas treated with intravitreal gene therapy aimed at expressing the type I opsin, bReaChES under the expression of the calcium calmodulin II alpha kinase promoter.^32^^,^^40^ First, we measured optogenetically evoked spike counts across a range of stimulus durations and estimated their SNR by quantifying the stimulus-driven increase in spiking events relative to the magnitude and variability of ongoing spontaneous activity. Next, we repeated the protocol in the presence of MFA. As shown in Figure 6, SNR increased monotonically with stimulus duration in both conditions, but was significantly increased in the presence of MFA. Across all pulse durations tested, MFA significantly enhanced the SNR relative to control, indicating that blockade of spontaneous hyperactivity via gap junction inhibition improves the detectability of light-evoked responses.

These findings suggest that suppression of spontaneous bursts leads to better discrimination between evoked and spontaneous activity. This improvement can be attributed to decreases in both background spike rate and burst-associated noise, thus enhancing response fidelity.

## Discussion

A central finding of our study is that the Rho-iCre-DTA176 mouse retina exhibits robust spontaneous rhythmic activity that closely mirrors the aberrant spiking phenotypes described in established models of retinal degeneration, including rd_1_ and rd_10_ mice. Retinal ganglion cells displayed elevated spontaneous firing rates, pronounced bursting, and a characteristic compression of interspike intervals, indicating that the emergence of pathological rhythmicity is not restricted to mutations directly affecting phototransduction cascades, as observed in the rd_1_ and rd_10_ models. Instead, these results support the broader view that loss of photoreceptor input precipitates a stereotyped form of inner retinal network remodeling that converges on similar functional outcomes across degeneration models.^22^^,^^31^^,^^41^

Importantly, the spontaneous activity observed here was not uniform tonic hyperactivity but instead exhibited clear temporal structure, with bursts recurring at quasi-regular intervals and dominating the spike statistics. This pattern aligns with previous reports showing that aberrant activity in degenerate retinas is temporally organized rather than random noise, a property that has significant consequences for downstream signal processing.^42^^,^^43^ Thus the Rho-iCre-DTA176 model faithfully reproduces a defining functional hallmark of retinal degeneration.

### Network-Driven Origins of Aberrant RGC Activity

Our findings are consistent with previous evidence that aberrant spiking in degenerating retinas is driven predominantly by presynaptic network activity rather than changes in intrinsic RGC excitability. Although firing rate and burst structure were altered, spontaneous spiking persisted after pharmacological suppression of network coupling and lacked features of intrinsic pacemaker behavior. This is consistent with studies indicating that rhythmic ganglion cell activity in degenerating retina arises from rhythmic synaptic input generated by a gap-junction coupled network of AII amacrine and ON cone bipolar cells, rather than from independent oscillatory currents in RGCs themselves.^28^^,^^44^^,^^45^

After gap-junction blockade, spiking persisted outside of bursts, arguing against a mechanism in which RGCs are silenced and instead supporting the decoupling of RGCs from a synchronizing presynaptic oscillator. Previous work shows that pharmacological or genetic disruption of gap junctions within the AII amacrine/ON cone bipolar cell network abolishes rhythmic bursting in RGCs while leaving residual, irregular spiking intact.^42^^,^^46^ These results support a model in which pathological RGC activity reflects emergent dynamics of electrically coupled retinal microcircuits that shape the output of surviving RGCs.

### Gap-Junction Coupling Is Critical for Rhythmic Bursting

A major mechanistic insight from this study is the pronounced sensitivity of spontaneous rhythmic activity to gap-junction blockade with MFA. MFA reliably abolished bursting and strongly reduced short-interval spiking, shifting ISI distributions toward longer intervals. This effect closely parallels observations in rd_1_ and rd_10_ retinas, where electrical coupling, particularly via Cx36-containing gap junctions, has been shown to be essential for sustaining oscillatory activity.^31^^,^^42^

The observed ISI redistribution after MFA provides important mechanistic constraints. A selective loss of short ISIs, rather than a uniform reduction in firing, suggests that gap junctions are specifically required for the generation or propagation of tightly clustered spikes that define bursts. This is consistent with models in which electrically coupled AII amacrine cells form a resonant network capable of generating rhythmic depolarizations that entrain downstream bipolar and RGCs.^28^ In this scenario, MFA disrupts temporal coherence within the network without abolishing all synaptic drive and abolishes burst expression rather than fully suppressing spiking activity.

At the same time, it should be noted that MFA is not selective for Cx36 and may exert additional effects on other conductances. However, the qualitative agreement between our findings and those obtained using genetic ablation of Cx36 strongly suggests that electrical coupling remains the main factor responsible for the observed effects.^42^

### Comparison With Established Retinal Degeneration Models

Comparison of spontaneous spiking properties highlights the close match between the Rho-iCre-DTA176 phenotype and established models of retinal degeneration but also reveals differences in magnitude and expression. In our recordings, dominant oscillatory activity in Rho-iCre-DTA176 retinas was concentrated in the low-frequency range (∼3–5 Hz), consisting of short burst durations (∼100 ms), and high intraburst firing rates, consistent with tightly clustered spike superimposed on a slower network rhythm. These values overlap substantially with those reported for rd_10_ retinas, which typically exhibit slower, less stereotyped oscillations in the ∼3–7 Hz range and longer, more variable bursts, in contrast to the faster (∼8–15 Hz), more rigidly phase-locked bursting observed in rd_1_.^22^^,^^28^^,^^31^ The lower oscillation frequency observed here likely reflects differences in the time course of degeneration and residual circuit integrity, rather than a distinct mechanism, given that evidence across models implicates a shared AII amacrine–ON cone bipolar oscillator whose operating regime is shaped by coupling strength, membrane excitability, and developmental stage. Importantly, despite variability in absolute frequencies and burst durations across studies, the key features of this aberrant activity appears conserved. These similarities indicate that spontaneous RGC bursting in retinal degeneration arises from a shared network mechanism that shapes how pathological noise constrains retinal output and degrades the fidelity of restored visual signals.

### Consequences of Aberrant Activity for Noise and Signal Fidelity

Spontaneous rhythmic activity has profound implications for retinal signal processing beyond its mechanistic significance. The temporal structure of bursting activity may overlap with the time course of physiologically evoked responses, rendering spontaneous and stimulus evoked spikes difficult to distinguish at the level of downstream decoders. Previous work has demonstrated that such overlap degrades signal-to-noise ratios and reduces the reliability of visual responses in degenerate retinas.^43^^,^^46^ Our observation that MFA reduces burst-associated short ISIs, and restores basic firing statistics toward control values, is therefore consistent with the idea that suppressing network oscillations can partially restore functional separability between spontaneous and evoked activity.

Importantly, these results highlight that the pathological impact of aberrant activity is not only limited to elevated firing rates but in the specific temporal organization of spikes. Bursting generates additional noise in retinal output, constituting a source of signal distortion that is particularly detrimental for optogenetic or prosthetic strategies that rely on temporal coding. Thus, targeting the mechanisms that generate rhythmicity may be as critical as restoring photoreceptor-like input itself.

### Comparison With Other Degeneration Models

The similarities between the Rho-iCre-DTA176 phenotype and those observed in classical degeneration models reinforce the generality of aberrant oscillatory activity as a consequence of photoreceptor loss. Notably, rhythmic spiking with comparable frequencies and burst statistics has also been observed after partial photoreceptor bleaching in otherwise healthy retinas,^45^ suggesting that the emergence of oscillations reflects the unmasking of latent circuit dynamics normally suppressed by photoreceptor dark current. In this sense, degeneration may not create a novel pathological circuit but rather reveal intrinsic network properties that become dominant in the absence of outer retinal input.

At the same time, quantitative differences across models, such as oscillation frequency, degree of synchrony, and spatial coherence (not discussed in our study), are likely to reflect differences in degeneration time course, residual photoreceptor input, and the extent of circuit remodeling. The Rho-iCre-DTA176 model therefore offers a valuable addition to the existing repertoire of retina degeneration models, enabling comparisons across degeneration mechanisms while preserving a common functional phenotype.

An important difference of the Rho-iCre-DTA176 model is the time course, rate and severity of photoreceptor degeneration relative to classical rd_1_ and rd_10_ mice. In rd_1_, rod degeneration begins at approximately P10-P14 and progresses rapidly, with near-complete rod loss and collapse of the outer nuclear layer by ∼P21.^9^^,^^47^ However, despite this profound structural degeneration, surviving cone somata and peripheral rod nuclei persist beyond this stage.^48^ Rod nuclei can still be detected in the far peripheral retina for several weeks after P21, reflecting a center-to-periphery gradient of degeneration, while cone photoreceptors exhibit substantially prolonged survival.^47^^,^^48^ In fact, cone cell bodies persist well beyond the period of outer segment loss, with substantial populations remaining at early postnatal stages and pronounced regional preservation in the peripheral retina, while a smaller residual population persists into adulthood.^47^^,^^48^ Consistent with this, cone outer segment loss around P21 leads to a marked reduction in ERG responses but residual photoreceptor-driven signaling may persist under certain conditions or strain backgrounds.^49^ In contrast, rd_10_ exhibits a delayed onset (∼P16–P18) and a more gradual degeneration extending to ∼P35–P45.^16^^,^^50^ By comparison, the Rho-iCre-DTA176 retina shows no detectable ERG responses and absence of PNA-positive cone outer segments already at three weeks,^32^ indicating an earlier and more profound loss of photoreceptor structure and function. This rapid, near-complete ablation provides a model in which inner retinal circuitry can be examined in a state largely devoid of photoreceptor input. Moreover, because degeneration in this model is not driven by PDE6B mutations, it avoids gene-specific effects associated with cyclic nucleotide dysregulation and instead provides a model for investigating mutation-independent mechanisms of retinal remodeling. As such, the Rho-iCre-DTA176 model complements rd_1_ and rd_10_ by allowing the study of common downstream circuit dysfunction and by providing a robust benchmark for evaluating restoration strategies that must operate across diverse genetic etiologies.

### Implications for Therapeutic Strategies

Taken together, these findings establish the Rho-iCre-DTA176 mouse as a valid model for studying spontaneous aberrant activity in retinal degeneration. The preservation of viable but pathologically driven RGCs highlights the potential for therapeutic interventions aimed at restoring stimulus induced activity in output neurons rather than replacing them. Pharmacological or genetic strategies that reduce electrical coupling, dampen oscillatory drive, or decorrelate spontaneous activity may substantially improve the efficacy of vision restoration approaches, including optogenetics, electrical stimulation and cell replacement therapies. More broadly, our work reinforces the idea that successful treatment of retinal degeneration must deal not only with loss of photoreceptor input but also with the emergent properties of the remodeled inner retina.

## References

[1] Hartong DT, Berson EL, Dryja TP. Retinitis pigmentosa. *Lancet*. 2006; 368(9549): 1795–1809. 17113430 10.1016/S0140-6736(06)69740-7

[2] Schneider N, Sundaresan Y, Gopalakrishnan P, et al. Inherited retinal diseases: linking genes, disease-causing variants, and relevant therapeutic modalities. *Prog Retin Eye Res*. 2022; 89: 101029. 34839010 10.1016/j.preteyeres.2021.101029

[3] Gill JS, Georgiou M, Kalitzeos A, Moore AT, Michaelides M. Progressive cone and cone-rod dystrophies: clinical features, molecular genetics and prospects for therapy. *Br J Ophthalmol*. 2019; 103: 711–720. 30679166 10.1136/bjophthalmol-2018-313278 PMC6709772

[4] Dias MF, Joo K, Kemp JA, et al. Molecular genetics and emerging therapies for retinitis pigmentosa: basic research and clinical perspectives. *Prog Retin Eye Res*. 2018; 63: 107–131. 29097191 10.1016/j.preteyeres.2017.10.004

[5] Broadgate S, Yu J, Downes SM, Halford S. Unravelling the genetics of inherited retinal dystrophies: past, present and future. *Prog Retin Eye Res*. 2017; 59: 53–96. 28363849 10.1016/j.preteyeres.2017.03.003

[6] Pennesi ME, Neuringer M, Courtney RJ. Animal models of age related macular degeneration. *Mol Aspects Med*. 2012; 33: 487–509. 22705444 10.1016/j.mam.2012.06.003 PMC3770531

[7] Schnichels S, Paquet-Durand F, Loscher M, et al. Retina in a dish: cell cultures, retinal explants and animal models for common diseases of the retina. *Prog Retin Eye Res*. 2021; 81: 100880. 32721458 10.1016/j.preteyeres.2020.100880

[8] Slijkerman RW, Song F, Astuti GD, et al. The pros and cons of vertebrate animal models for functional and therapeutic research on inherited retinal dystrophies. *Prog Retin Eye Res*. 2015; 48: 137–159. 25936606 10.1016/j.preteyeres.2015.04.004

[9] Bowes C, Li T, Danciger M, Baxter LC, Applebury ML, Farber DB. Retinal degeneration in the rd mouse is caused by a defect in the beta subunit of rod cGMP-phosphodiesterase. *Nature*. 1990; 347(6294): 677–680. 1977087 10.1038/347677a0

[10] Hart AW, McKie L, Morgan JE, et al. Genotype–phenotype correlation of mouse Pde6b mutations. *Invest Ophthalmol Vis Sci*. 2005; 46: 3443–3450. 16123450 10.1167/iovs.05-0254

[11] Mandai M, Fujii M, Hashiguchi T, et al. iPSC-Derived retina transplants improve vision in rd1 end-stage retinal-degeneration mice. *Stem Cell Rep*. 2017; 8: 69−83. 10.1016/j.stemcr.2016.12.008 PMC5233464 28076757

[12] Singh MS, Charbel Issa P, Butler R, et al. Reversal of end-stage retinal degeneration and restoration of visual function by photoreceptor transplantation. *Proc Natl Acad Sci*. 2013; 110: 1101–1106. 23288902 10.1073/pnas.1119416110 PMC3549087

[13] Vighi E, Trifunović D, Veiga-Crespo P, et al. Combination of cGMP analogue and drug delivery system provides functional protection in hereditary retinal degeneration. *Proc Natl Acad Sci*. 2018; 115(13): E2997–E3006. 29531030 10.1073/pnas.1718792115 PMC5879685

[14] Luodan A, Zou T, He J, et al. Rescue of retinal degeneration in rd1 mice by intravitreally injected metformin. *Front Mol Neurosci*. 2019; 12: 102. 31080404 10.3389/fnmol.2019.00102 PMC6497809

[15] Noben-Trauth K, Naggert JK, North MA, Nishina PM. A candidate gene for the mouse mutation tubby. *Nature*. 1996; 380(6574): 534–538. 8606774 10.1038/380534a0

[16] Chang B, Hawes NL, Hurd RE, Davisson MT, Nusinowitz S, Heckenlively JR. Retinal degeneration mutants in the mouse. *Vis Res*. 2002; 42: 517–525. 11853768 10.1016/s0042-6989(01)00146-8

[17] Lai YL, Jacoby RO, Jonas AM. Age-related and light-associated retinal changes in Fischer rats. *Invest Ophthalmol Vis Sci*. 1978; 17: 634–638. 669893

[18] Strauss O, Stumpff F, Mergler S, Wienrich M, Wiederholt M. The Royal College of Surgeons rat: an animal model for inherited retinal degeneration with a still unknown genetic defect. *Acta Anat*. 1998; 162(2-3): 101–111. 9831756 10.1159/000046474

[19] Collin GB, Gogna N, Chang B, et al. Mouse models of inherited retinal degeneration with photoreceptor cell loss. *Cells*. 2020; 9: 931. 32290105 10.3390/cells9040931 PMC7227028

[20] Hafezi F, Grimm C, Simmen BC, Wenzel A, Reme CE. Molecular ophthalmology: an update on animal models for retinal degenerations and dystrophies. *Br J Ophthalmol*. 2000; 84: 922–927. 10906106 10.1136/bjo.84.8.922 PMC1723576

[21] Margolis DJ, Detwiler PB. Cellular origin of spontaneous ganglion cell spike activity in animal models of retinitis pigmentosa. *J Ophthalmol*. 2011; 2011: 507037. 20936060 10.1155/2011/507037 PMC2948917

[22] Stasheff SF. Emergence of sustained spontaneous hyperactivity and temporary preservation of OFF responses in ganglion cells of the retinal degeneration (rd1) mouse. *J Neurophysiol*. 2008; 99: 1408–1421. 18216234 10.1152/jn.00144.2007

[23] Euler T, Schubert T. Multiple independent oscillatory networks in the degenerating retina. *Front Cell Neurosci*. 2015; 9: 444. 26617491 10.3389/fncel.2015.00444 PMC4637421

[24] Zeck G. Aberrant activity in degenerated retinas revealed by electrical imaging. *Front Cell Neurosci*. 2016; 10: 25. 26903810 10.3389/fncel.2016.00025 PMC4758270

[25] Telias M, Nawy S, Kramer RH. Degeneration-dependent retinal remodeling: looking for the molecular trigger. *Front Neurosci*. 2020; 14: 618019. 33390897 10.3389/fnins.2020.618019 PMC7775662

[26] Trenholm S, Awatramani GB. Origins of spontaneous activity in the degenerating retina. *Front Cell Neurosci*. 2015; 9: 277. 26283914 10.3389/fncel.2015.00277 PMC4518194

[27] Barrett JM, Degenaar P, Sernagor E. Blockade of pathological retinal ganglion cell hyperactivity improves optogenetically evoked light responses in rd1 mice. *Front Cell Neurosci*. 2015; 9: 330. 26379501 10.3389/fncel.2015.00330 PMC4548307

[28] Trenholm S, Borowska J, Zhang J, et al. Intrinsic oscillatory activity arising within the electrically coupled AII amacrine-ON cone bipolar cell network is driven by voltage-gated Na+ channels. *J Physiol*. 2012; 590: 2501–2517. 22393249 10.1113/jphysiol.2011.225060 PMC3424767

[29] Vaney DI, Nelson JC, Pow DV. Neurotransmitter coupling through gap junctions in the retina. *J Neurosci*. 1998; 18: 10594. 9852595 10.1523/JNEUROSCI.18-24-10594.1998 PMC6793342

[30] Vardi N, Smith RG. The AII Amacrine Network: coupling can increase correlated activity. *Vis Res*. 1996; 36: 3743–3757. 8994576 10.1016/0042-6989(96)00098-3

[31] Menzler J, Zeck G. Network oscillations in rod-degenerated mouse retinas. *J Neurosci*. 2011; 31: 2280–2291. 21307264 10.1523/JNEUROSCI.4238-10.2011 PMC6633031

[32] Too LK, Shen W, Protti DA, et al. Optogenetic restoration of high sensitivity vision with bReaChES, a red-shifted channelrhodopsin. *Sci Rep*. 2022; 12(1): 19312. 36369267 10.1038/s41598-022-23572-4 PMC9652428

[33] Li S, Chen D, Sauve Y, McCandless J, Chen YJ, Chen CK. Rhodopsin-iCre transgenic mouse line for Cre-mediated rod-specific gene targeting. *Genesis*. 2005; 41: 73–80. 15682388 10.1002/gene.20097

[34] Shen W, Fruttiger M, Zhu L, et al. Conditional Mueller cell ablation causes independent neuronal and vascular pathologies in a novel transgenic model. *J Neurosci*. 2012; 32: 15715–15727. 23136411 10.1523/JNEUROSCI.2841-12.2012 PMC4014009

[35] De Silva SR, Barnard AR, Hughes S, et al. Long-term restoration of visual function in end-stage retinal degeneration using subretinal human melanopsin gene therapy. *Proc Natl Acad Sci U S A*. 2017; 114: 11211–11216. 28973921 10.1073/pnas.1701589114 PMC5651734

[36] Pachitariu M, Steinmetz N, Kadir S, Carandini M, Kenneth DH. Kilosort: realtime spike-sorting for extracellular electrophysiology with hundreds of channels. *bioRxiv*. Preprint posted online June 30, 2016. doi:10.1101/061481.

[37] Pachitariu M, Steinmetz NA, Kadir SN, Carandini M, Harris KD. Fast and accurate spike sorting of high-channel count probes with KiloSort. *Presented at: Advances in Neural Information Processing Systems 29*; 2016 Barcelona, Spain.

[38] Burns T, Rajan R. A mathematical approach to correlating objective spectro-temporal features of non-linguistic sounds with their subjective perceptions in humans. *Front Neurosci*. 2019; 13: 794. 31417350 10.3389/fnins.2019.00794 PMC6685481

[39] Tchernichovski O, Nottebohm F, Ho CE, Pesaran B, Mitra PP. A procedure for an automated measurement of song similarity. *Anim Behav*. 2000; 59: 1167–1176. 10877896 10.1006/anbe.1999.1416

[40] Rajasethupathy P, Sankaran S, Marshel JH, et al. Projections from neocortex mediate top-down control of memory retrieval. *Nature*. 2015; 526(7575): 653–659. 26436451 10.1038/nature15389 PMC4825678

[41] Margolis DJ, Newkirk G, Euler T, Detwiler PB. functional stability of retinal ganglion cells after degeneration-induced changes in synaptic input. *J Neurosci*. 2008; 28: 6526–6536. 18562624 10.1523/JNEUROSCI.1533-08.2008 PMC3050548

[42] Ivanova E, Yee CW, Baldoni R Jr., Sagdullaev BT. Aberrant activity in retinal degeneration impairs central visual processing and relies on Cx36-containing gap junctions. *Exp Eye Res*. 2016; 150: 81–89. 26005040 10.1016/j.exer.2015.05.013 PMC4655183

[43] Margolis DJ, Gartland AJ, Singer JH, Detwiler PB. Network oscillations drive correlated spiking of ON and OFF ganglion cells in the rd1 mouse model of retinal degeneration. *PLoS One*. 2014; 9(1): e86253. 24489706 10.1371/journal.pone.0086253 PMC3904909

[44] Choi H, Zhang L, Cembrowski MS, et al. Intrinsic bursting of AII amacrine cells underlies oscillations in the rd1 mouse retina. *J Neurophysiol*. 2014; 112: 1491–1504. 25008417 10.1152/jn.00437.2014 PMC4137253

[45] Menzler J, Channappa L, Zeck G. Rhythmic ganglion cell activity in bleached and blind adult mouse retinas. *PLoS One*. 2014; 9(8): e106047. 25153888 10.1371/journal.pone.0106047 PMC4143350

[46] Toychiev AH, Ivanova E, Yee CW, Sagdullaev BT. Block of gap junctions eliminates aberrant activity and restores light responses during retinal degeneration. *J Neurosci*. 2013; 33: 13972–13977. 23986234 10.1523/JNEUROSCI.2399-13.2013 PMC3756747

[47] Carter-Dawson LD, LaVail MM, Sidman RL. Differential effect of the rd mutation on rods and cones in the mouse retina. *Invest Ophthalmol Vis Sci*. 1978; 17: 489–498. 659071

[48] Narayan DS, Ao J, Wood JPM, Casson RJ, Chidlow G. Spatio-temporal characterization of S- and M/L-cone degeneration in the Rd1 mouse model of retinitis pigmentosa. *BMC Neurosci*. 2019; 20: 46. 31481030 10.1186/s12868-019-0528-2 PMC6720080

[49] van Wyk M, Schneider S, Kleinlogel S. Variable phenotypic expressivity in inbred retinal degeneration mouse lines: a comparative study of C3H/HeOu and FVB/N rd1 mice. *Mol Vis*. 2015; 21: 811–827. 26283863 PMC4522243

[50] Gargini C, Terzibasi E, Mazzoni F, Strettoi E. Retinal organization in the retinal degeneration 10 (rd10) mutant mouse: a morphological and ERG study. *J Comp Neurol*. 2007; 500: 222–238. 17111372 10.1002/cne.21144 PMC2590657

